# Assessment of Reporting Completeness in Prostate Core Biopsies: A Four-Year Audit From a Tertiary Care Centre (2021-2024)

**DOI:** 10.7759/cureus.104516

**Published:** 2026-03-01

**Authors:** Asim Qureshi, Alanood Al Sulaimani, Hannia Qureshi, Marwa Al Riyami

**Affiliations:** 1 Pathology, Sultan Qaboos University Hospital, Muscat, OMN; 2 Histopathology, Oman Medical Speciality Board, Muscat, OMN; 3 Medicine, Combined Military Hospital Multan, Multan, PAK

**Keywords:** audit, biopsy, histopathology reporting, prostate biopsy, prostate cancer, urology surgery

## Abstract

Background: Accurate and standardised histopathology reporting of prostate core biopsies is essential for appropriate risk stratification, prognostication, and clinical decision-making in patients with prostatic adenocarcinoma. Adherence to internationally recognised reporting guidelines ensures consistency, completeness, and clinical relevance of pathology reports. Clinical audits serve as an effective quality assurance tool to evaluate reporting practices and identify areas requiring improvement.

Objective: This study aimed to assess the completeness of histopathological reporting of prostate core biopsies diagnosed as prostatic adenocarcinoma at a tertiary care academic hospital, using established international standards as audit benchmarks.

Methods: A retrospective clinical audit was conducted in the Histopathology Department of Sultan Qaboos University Hospital. Prostate core biopsy reports issued between January 2021 and December 2024 were retrieved from the laboratory information system. A total of 116 consecutive prostate biopsies were included, comprising both benign and malignant diagnoses. Reporting completeness was evaluated only in the 64 cases diagnosed as prostatic adenocarcinoma. Audit criteria were derived from the College of American Pathologists (CAP) Prostate Cancer Protocol (2023 update), the WHO Classification of Tumours of the Urinary System and Male Genital Organs (2022), and the International Society of Urological Pathology (ISUP) consensus guidelines on Gleason grading. Key parameters assessed included histological tumour type, primary and secondary Gleason patterns, total Gleason score, WHO/ISUP Grade Group, perineural invasion status, tumour percentage per core, and documentation of tissue fragment count. Data were analysed descriptively using counts and proportions.

Results: Of the 116 prostate biopsies reviewed, 52 cases (45%) were benign, and 64 cases (55%) were diagnosed as prostatic adenocarcinoma. All malignant cases (100%) had complete documentation of Gleason grading parameters, reflecting excellent compliance with ISUP recommendations. Histological tumour type was reported in 94% of cases, WHO/ISUP Grade Group in 95%, and perineural invasion status in 97%. Tumour percentage per core was documented in 92% of cases, representing the most frequently omitted parameter. Gross description, including tissue fragment count, was complete in all cases.

Conclusion: This audit demonstrates a high level of adherence to international prostate biopsy reporting standards, with minor deficiencies identified in the reporting of tumour extent. Regular audits and targeted interventions may further enhance reporting quality and standardisation.

## Introduction

Prostate cancer is one of the leading malignancies affecting men worldwide and represents a significant public health burden both globally and regionally. According to the Global Cancer Observatory (GLOBOCAN) 2022 data, prostate cancer is the second most frequently diagnosed cancer in men and a major contributor to cancer-related mortality [1].

Early and accurate diagnosis is essential for guiding effective management strategies, which may include active surveillance, radical prostatectomy, radiotherapy, or hormone therapy. Prostate core biopsy is the definitive diagnostic method, providing histological confirmation of malignancy and evaluation of tumour grade and burden [2]. Key information derived from biopsy specimens - particularly the Gleason score, WHO Grade Group, tumour percentage, and perineural invasion (PNI) - plays an integral role in patient management algorithms, such as the National Comprehensive Cancer Network (NCCN) and European Association of Urology (EAU) risk-stratification models.

The consistency and completeness of histopathology reporting are critical given the importance of biopsy results in clinical decision-making. International organisations, including the College of American Pathologists (CAP), the International Society of Urological Pathology (ISUP), and the World Health Organisation (WHO), have published guidelines outlining the essential elements that should be included in prostate biopsy reports. Increasingly, synoptic reporting formats are encouraged to reduce variability, enhance completeness, and improve communication with clinicians [3,4]. This audit is part of the audit cycle and will be followed by a reaudit. It also presents a framework for further work to improve reporting quality.

Clinical audits are valuable tools for assessing adherence to reporting standards and identifying areas for quality improvement. While several audits have been conducted globally, data from the Middle East, including Oman, is limited. There is a lack of published audits of prostate biopsy reporting in Oman. The importance of regional data for quality assurance and benchmarking cannot be undermined. This study provides a comprehensive assessment of prostate biopsy reporting at Sultan Qaboos University Hospital (SQUH) over the past four years. To our knowledge, this is the first comprehensive audit of prostate core biopsies from Oman and would provide a framework for further work.

## Materials and methods

Study design

A retrospective clinical audit was conducted at the Histopathology Department of a primary tertiary care and academic hospital. The audit period covered January 2021 through December 2024.

Study population and sample size

A total of 116 consecutive prostate core biopsy reports were reviewed, including benign and malignant diagnoses. These were continuous samples from patients who came to the university hospital for prostatic symptoms and underwent prostatic core biopsies. Only the 64 biopsies diagnosed as prostatic adenocarcinoma were assessed for reporting completeness. All biopsies were transurethral ultrasound-guided and obtained by the same surgeon. Twelve biopsies were taken from each patient. An IRB approval was obtained for the study from the College of Medicine & Health Sciences, Sultan Qaboos University (SQU-EC/299/2023MREC#3185).

Study measures

Audit criteria were based on internationally recognised reporting standards, including the CAP Prostate Cancer Protocol (2023 update), the WHO Classification of Tumours of the Urinary System and Male Genital Organs (2022), and the ISUP Consensus Guidelines on Gleason grading (2014, updated 2019).

The following key parameters were evaluated in each malignant case: histological tumour type, primary and secondary Gleason patterns, Gleason score, WHO/ISUP Grade Group, PNI status, tumour percentage per core, and gross description (number of tissue fragments/cores).

Each parameter was categorised as “reported” or “not reported” according to the CAP, WHO, and ISUP minimum dataset recommendations. Expected compliance was 100% completeness; this was the institutional goal.

Statistical analysis

Data were summarised descriptively using counts and proportions. No inferential statistics (a branch of statistics that uses data analysed from a small sample to make inferences, predictions, or generalisations about a larger population) were applied, as the primary aim was quality assessment rather than hypothesis testing.

## Results

A total of 116 prostate core biopsies were reviewed, of which 52 cases (45%) were benign, and 64 cases (55%) showed prostatic adenocarcinoma. Evaluation of reporting completeness in malignant cases demonstrated a high level of adherence to recommended histopathological reporting standards. Histological tumour type was documented in 60 of 64 cases (94%), with omission in four cases. Gleason grading parameters, including primary grade, secondary grade, and total Gleason score, were reported in all malignant cases (100%), reflecting excellent compliance with ISUP grading guidelines. The WHO/ISUP Grade Group was included in 61 cases (95%), while PNI status was documented in 62 cases (97%). Tumour involvement per core, expressed as tumour percentage, was reported in 59 cases (92%), representing the most frequently omitted parameter. Documentation of the number of core fragments in the gross description was complete in all 116 cases (100%), including benign biopsies, indicating consistent grossing practices across the cohort (Table 1).

**Table 1:** Completeness of Reporting in Prostate Core Biopsy Cases (n = 116) WHO: World Health Organisation; ISUP: the International Society of Urological Pathology

Parameter	Number of Cases Reported	Total Applicable Cases	Percentage (%)
Overall Case Distribution			
Benign diagnoses	52	116	45
Malignant diagnoses (adenocarcinoma)	64	116	55
Reporting Parameters in Malignant Cases (n = 64)			
Histological tumour type	60	64	94
Primary Gleason grade	64	64	100
Secondary Gleason grade	64	64	100
Total Gleason score	64	64	100
WHO/ISUP Grade Group	61	64	95
Perineural invasion status	62	64	97
Tumour percentage per core	59	64	92
Gross Description (All Cases)			
Core fragment count documented	116	116	100

## Discussion

This four-year audit demonstrates a high standard of reporting for prostate biopsies at SQUH, particularly regarding Gleason grading, which achieved 100% completeness. This aligns with global best practices and reflects the department’s strong diagnostic competency. However, notable gaps were observed in a few reporting parameters, specifically tumour percentage per core, PNI, and Grade Group [3].

Implementation of synoptic reporting templates with a defined timeline for re-audit (e.g., at 12 months) may be required. The Gleason system remains the cornerstone of prostate cancer grading and is one of the strongest prognostic indicators in localised disease. The introduction of the WHO/ISUP Grade Group system in 2014, with updates in the 2022 WHO classification, simplified prognostication and improved communication with clinicians. Missing Grade Group data, even in a small number of cases, can create ambiguity in clinical decision-making [3].

Tumour percentage per core is a significant determinant of eligibility for active surveillance. Studies have shown that tumour burden correlates strongly with biochemical recurrence and adverse pathological outcomes at prostatectomy. The missing tumour percentage in five cases represents a clinically meaningful gap, as incomplete documentation may directly affect treatment recommendations [5].

PNI is a well-recognised histopathological feature with prognostic and therapeutic implications. Although its independent prognostic role remains debated, it is frequently used in risk-stratification algorithms [6]. The absence of PNI documentation in two cases suggests the need for a checklist-based reminder system or template [7].

Narrative reporting may offer stylistic flexibility but is prone to omitting critical details. Synoptic reporting templates, widely used in Australia, Canada, and the U.S., have consistently improved reporting completeness, data extraction, and multidisciplinary team (MDT) clarity. Integrating synoptic templates through the laboratory information system at SQUH would likely eliminate the omissions observed in this audit [7].

The comprehensive review conducted over four years serves as a critical examination of current practices, ensuring alignment with international standards and enhancing the credibility and relevance of the findings. Throughout this period, the focus has been on clearly identifying actionable gaps, which facilitates targeted interventions and improvements in clinical processes. However, the audit was limited to a single centre, which may limit the generalizability of the results across diverse settings, though it allowed for a more in-depth analysis of the institution's practices. To enhance the quality and accuracy of pathology reporting, implementing a mandatory synoptic reporting template embedded within the digital reporting system is essential. This standardised approach can facilitate clear, consistent documentation. Additionally, conducting regular audits, ideally annually, will help monitor progress, identify areas for improvement, and maintain high standards of practice.

Limitations

This is a single-centre study, and the number of patients is small; however, the study's findings can be generalised to the national level, as this centre is a tertiary care facility that receives a significant number of prostate cancer patients.

## Conclusions

This four-year audit shows that prostate biopsy reporting at SQUH meets high international standards in key diagnostic areas, particularly Gleason grading. However, variability in reporting tumour percentage, WHO Grade Group, and PNI indicates opportunities for quality improvement. Adopting structured synoptic reporting and maintaining regular audits will enhance consistency, clinical value, and adherence to global best practices. Ultimately, these improvements will help ensure optimal patient care and more accurate risk stratification for men with prostate cancer in Oman.
